# Drivers of Tuberculosis Transmission

**DOI:** 10.1093/infdis/jix354

**Published:** 2017-11-03

**Authors:** Barun Mathema, Jason R Andrews, Ted Cohen, Martien W Borgdorff, Marcel Behr, Judith R Glynn, Roxana Rustomjee, Benjamin J Silk, Robin Wood

**Affiliations:** 1 Department of Epidemiology, Mailman School of Public Health, Columbia University, New York, New York;; 2 Division of Infectious Diseases and Geographic Medicine, Stanford University, California;; 3 Department of Epidemiology of Microbial Diseases, Yale School of Public Health, New Haven, Connecticut;; 4 Centers for Disease Control and Prevention, Kisumu, Kenya;; 5 Department of Clinical Epidemiology, Biostatistics, and Bioinformatics, Academic Medical Center, University of Amsterdam, the Netherlands;; 6 McGill International TB Centre, Research Institute of the McGill University Health Centre, Montreal,Canada;; 7 Faculty of Epidemiology and Population Health, London School of Hygiene and Tropical Medicine, United Kingdom;; 8 Tuberculosis Clinical Research Branch, Therapeutics Research Program, Division of AIDS, National Institute of Allergy and Infectious Diseases, National Institutes of Health, Department of Health and Human Services, Rockville, Maryland;; 9 Division of Tuberculosis Elimination, Centers for Disease Control and Prevention, Atlanta, Georgia;; 10 Desmond Tutu HIV Centre, Institute of Infectious Disease and Molecular Medicine, University of Cape Town, South Africa

**Keywords:** tuberculosis, transmission, epidemiology, estimating transmission, transmission amplifiers

## Abstract

Measuring tuberculosis transmission is exceedingly difficult, given the remarkable variability in the timing of clinical disease after *Mycobacterium tuberculosis* infection; incident disease can result from either a recent (ie, weeks to months) or a remote (ie, several years to decades) infection event. Although we cannot identify with certainty the timing and location of tuberculosis transmission for individuals, approaches for estimating the individual probability of recent transmission and for estimating the fraction of tuberculosis cases due to recent transmission in populations have been developed. Data used to estimate the probable burden of recent transmission include tuberculosis case notifications in young children and trends in tuberculin skin test and interferon γ–release assays. More recently, *M. tuberculosis* whole-genome sequencing has been used to estimate population levels of recent transmission, identify the distribution of specific strains within communities, and decipher chains of transmission among culture-positive tuberculosis cases. The factors that drive the transmission of tuberculosis in communities depend on the burden of prevalent tuberculosis; the ways in which individuals live, work, and interact (eg, congregate settings); and the capacity of healthcare and public health systems to identify and effectively treat individuals with infectious forms of tuberculosis. Here we provide an overview of these factors, describe tools for measurement of ongoing transmission, and highlight knowledge gaps that must be addressed.

Mounting evidence from countries with high tuberculosis burdens and varying prevalences of human immunodeficiency virus (HIV) infection points to ongoing transmission as the driving force maintaining tuberculosis incidence; this evidence appears to be even more pronounced for multidrug-resistant tuberculosis (MDR-TB) and extensively drug-resistant tuberculosis (XDR-TB) [1–3]. The intensity of local tuberculosis transmission is dependent on the prevalence (and infectiousness) of individuals with infectious forms of tuberculosis, the number (and susceptibility) of individuals with whom infectious individuals may contact, the frequency and proximity of interactions between infectious and susceptible individuals, as well as biological features of *Mycobacterium tuberculosis* (the pathogen) that affect transmission. Each of these factors can differ by setting; for example, the prevalence and infectiousness of individuals may be affected by individual-level attributes (eg, HIV coinfection) or by shared, community-level attributes (eg, accessibility of diagnosis and quality of care). Social-mixing patterns are influenced by age and demographic structure, cultural behaviors, population density, and migration patterns. Environmental factors, such as crowding and ventilation, have a direct impact on the person-to-person air exchange necessary for transmission. In addition, ways in which different strains of *M. tuberculosis* may be adapted for airborne survival, tuberculosis aerobiology, and successful infection are subjects of increasing research [4–6].

Measuring tuberculosis transmission and estimating the relative importance of recent transmission is exceedingly difficult, given that infection in only a minority of individuals will progress to disease and that the periods of latency among individuals with infection that do progress are variable. The infectiousness of tuberculosis cases can be defined clinically, including the presence of cough and acid-fast organisms in sputum, when individuals have not yet received effective tuberculosis therapy. However, infectiousness may be present prior to the onset of symptoms [7–9]. In fact, there is a spectrum in the clinical course of tuberculosis [10] inclusive of: subclinical tuberculosis (characterized by negative results of tuberculosis symptom screening but positive results of culture, with tuberculosis presumably infectious); prediagnostic disease (characterized by symptoms that are sufficiently noticeable for detection during symptom screening but not sufficiently severe to seek medical care); and clinical disease (characterized by active seeking of care for symptoms, although often after delays due to difficulties differentiating between other respiratory tract infections in settings where clinical diagnosis remains standard practice) [11]. Given these complexities, including the independent nature of these parameters, the relative contributions of highly infectious individuals versus apparently asymptomatic but infectious individuals in settings of high tuberculosis endemicity is uncertain.

This article, an output of a workshop entitled “How Do We Measure Tuberculosis Transmission Better,” discusses the main themes of the measurement of tuberculosis transmission and specific drivers of tuberculosis transmission, the characterization of infectious and susceptible individuals, and how we can use this information to model and halt tuberculosis transmission. The overarching goal of the workshop was to identify research gaps to address as a critical step in the development of novel interventions to stop tuberculosis transmission in high-incidence settings.

## FACTORS INFLUENCING TUBERCULOSIS TRANSMISSION

The probability that an individual with tuberculosis will transmit *M. tuberculosis* to others is determined by many factors. First, individuals with more severe pulmonary tuberculosis may emit higher numbers of infectious droplet nuclei by producing droplets at an elevated rate [12, 13]. The rate of droplet production may be affected by the frequency and vigor of coughing and by pathology-related factors that allow pathogens to escape into the airway (eg, cavities) [14]. Recent work suggests that measures of cough aerosol production may identify individuals more likely to contribute to community transmission [15, 16]. Closer proximity and longer duration of contact between an infectious source case and susceptible individuals increase the risk of transmission. Delays in the diagnosis of tuberculosis or initiation of adequate treatment increase the prevalence of infectious tuberculosis, thereby also increasing the probability of onward transmission [17, 18]. Contributions to treatment delay include individual healthcare-seeking behavior, structural barriers to healthcare access, and rapidity of diagnosis and treatment initiation within the health system.

Second, environmental factors that may increase the risk of infection include closed indoor spaces with limited air circulation and minimal UV light exposure, which provide an ideal environment for airborne particles containing *M. tuberculosis* to remain viable and infectious. Humidity also affects the settling and evaporation of droplet nuclei and thus likely affects the risk of infection [12].

Third, host factors may increase an individual’s risk of progression to active pulmonary disease after infection. Host-related determinants of disease risk include HIV infection [19], diabetes [20], smoking [21], excess alcohol use [22], and malnutrition [23]. The degree of infectiousness of the inoculum (ie, dose) also may influence the likelihood of disease [24]. Young children and infants, having both recent exposure by nature and a higher risk of rapid progression, can act as sentinel populations for ongoing transmission. However, they rarely contribute to ongoing transmission, owing to their decreased infectiousness [25, 26]. In older populations, incident tuberculosis is a less reliable measure of ongoing transmission because of difficulties in identifying the timing of infection and distinguishing between reactivation of latent infection and early active disease [10].

## MEASUREMENT OF TUBERCULOSIS TRANSMISSION

Four main approaches are used to measure tuberculosis transmission and identify its drivers. The first approach is case notification rates, which are used to identify countries and regions in which the risk of tuberculosis is high [27]. Notification rates also may yield temporal trends in transmission [28]; furthermore, they can be used to identify the heterogeneity of risk in subpopulations, such as a higher risk in low-incidence settings among foreign-born persons, prisoners, and those experiencing homelessness [29], as well as heterogeneity of risk among community locations within high-incidence settings (eg, public transportation [30], churches [31], schools [32, 33], bars [31], and slums [34–36]). Second, one can estimate the risk of being infected and identify risk factors for being infected. This approach uses the tuberculin skin test and interferon γ–release assays to identify infections. Estimates of trends in the annual risk of infection have been derived from repeated surveys, often including primary school children [37–39]. Repeated surveys have shown a higher risk of infection among adolescents than among children of primary school age [40, 41]. However, repeated cross-sectional studies are sensitive to prior changes in transmission at earlier ages, although reversion of tuberculin skin test and interferon γ–release assay findings may result in an underestimate of *M. tuberculosis* infection rates. Furthermore, in high transmission settings, repeat infection episodes cannot be easily identified with these types of infection assays that provide some measure of cumulative exposure. To estimate newly acquired infection, screening for *M. tuberculosis* infection can also be done in contact investigations and among high-risk groups, such as immigrants from high-burden countries or healthcare workers. Since molecular strain typing became available in the 1990s [42], a third approach, assessing the genotypic links between presumed index tuberculosis cases and their presumed secondary cases, has become possible. At the population level, methods such as *n* − 1 and other modeling-based techniques have been developed to estimate levels of recent transmission, based on genotypic clustering [43–46]. Molecular epidemiological studies have also confirmed that adolescents are a high-risk group for tuberculosis transmission, established the importance of nosocomial transmission [34], and identified other risk factors for recent transmission, including urban residence, homelessness [47], and exposure to crowded settings, such as prisons [48, 49]. Over time, the resolution of strain typing has improved; whole-genome sequencing (WGS) now provides the ultimate ability to distinguish strains on the basis of single nucleotide differences, as described in the next section below [50]. It is worth noting that the first and third methods depend in part on transmission and in part on the risk of progression from infection to disease. A fourth and indirect approach has been the characterization of source cases. Traditionally, measures of bacillary load based on sputum smear and the presence and extent of pulmonary cavitation have been used to estimate the infectiousness of source cases as a proxy for the expected number of infected contacts and secondary cases [51]. More recently, new measures of infectiousness have been developed [4, 6].

Other approaches to identify drivers of transmission have included contact surveys [32] and a recently developed method combining time and motion studies with measurement of CO_2_ in air as a measure of exhaled air [30]. These methods and their application and limitations are described in another article in this issue.

## ASSESSING TRANSMISSION BY USING MOLECULAR AND GENOMIC EPIDEMIOLOGICAL METHODS

Since the MDR-TB outbreaks in the 1990s in the United States, genotyping of *M. tuberculosis* isolates has been a critical element for studying tuberculosis transmission [44, 52]. A crucial aspect in understanding the transmission patterns of tuberculosis is the ability to track the frequency and spread of specific strains in the population [53]. Although previous genotyping methods (eg, IS*6110*-based restriction fragment–length polymorphism analysis and mycobacterial interspersed repetitive units variable number of tandem repeats analysis) are informative, these methods have limited discriminatory power. Newer methods, which are able to examine much more of the genome, suggest that some genotype matches between two patients’ isolates might not indicate recent transmission. Therefore, outbreaks confirmed by molecular methods in the 1990s and 2000s may have overestimated the number of cases that were truly part of a transmission chain [54]. The enhanced level of discrimination achieved by WGS is particularly important for characterizing the actual diversity of strains within a given lineage or genotype that appear to be closely related and for helping to rule out recent transmission when epidemiologic links have not been established [54]. The continual decline in costs, increased computing power, and ease of data sharing have facilitated access to WGS as a tool for investigating tuberculosis transmission.

Phylogenetic analysis using WGS data can detail strain relatedness at unprecedented levels of granularity (ie, single-nucleotide polymorphisms [SNPs]). Such data have been used to support estimates of levels of incident tuberculosis due to transmission in a variety of settings [1, 2, 54, 55]. A key question is: what levels of genetic variation are suggestive of transmission? The level of WGS data–based similarity (ie, the threshold of SNP differences) that is sufficient to consider two isolates related has proven difficult to establish. Most large-scale studies to date have used empirical SNP difference cutoffs, typically supported by epidemiological investigations [1, 55]. There are a number of factors to consider in determining levels of genetic variation in support of transmission. The average mutation rate in *M. tuberculosis* is estimated at 0.3–0.5 SNPs/genome/year between patient samples. However, considerable within-host variation has been noted in serial patient isolates, as well as in specimens from discrete environments of the lung [56–60]. The mutation rate also may vary on the basis of *M. tuberculosis* lineage or host characteristics, such as HIV coinfection [56–59]. More recently, methods to infer person-to-person transmission by using genomic information that consider within-host variation [61] and account for the complex and variable nature of case sampling (ie, undersampling) have been developed [62]. However, factors that impact heterogeneity are likely to be dependent on individual-level (eg, HIV status) and ecological-level (eg, force of infection) factors. A study by Chengalroyen et al details the presence of differentially culturable *M. tuberculosis* with mixed genotypes that vary by HIV status, a finding that warrants further investigation [63].

## IDENTIFYING AND DEFINING TUBERCULOSIS OUTBREAKS

Tuberculosis outbreaks typically present the clearest evidence of tuberculosis transmission, as they are often spatiotemporally restricted and involve shared epidemiological attributes. A first question is whether an unexpected number of tuberculosis cases, clustered by space and time, is indeed an outbreak. Epidemiologic studies have shown that tuberculosis does not afflict all susceptible individuals equally; therefore, an aggregation of individuals with risk factors for tuberculosis, such as in a hospital or prison, may actually represent a pseudo-outbreak. To establish that there has been *M. tuberculosis* spread from a source patient to multiple secondary cases, investigators can use molecular or genomic typing methods to demonstrate clonal dissemination.

By studying tuberculosis outbreaks defined by strain typing methods, it is possible to ask whether there are host, pathogen, and environmental factors significantly associated with recently transmitted disease. A caveat is that the study duration should account for *M. tuberculosis* latency (eg, a 1-year study cannot define risk factors for becoming infected and developing disease in 18 months). As such, molecular epidemiologic investigations of tuberculosis outbreaks do not measure transmission completely. Instead, they identify risk factors for the subset of new infections that rapidly progress to culture-confirmed tuberculosis. For defining a preventive intervention, this may be valuable information. However, such findings may be insufficient for understanding all of the determinants contributing to the original transmission events.

An important consideration when investigating risk factors for being a case in an outbreak defined by strain typing methods is whether the source is truly known. When grouping together index and secondary cases on the basis of a shared genotype, some individuals may have smear-negative pulmonary disease. These cases, which most likely reflect primary disease in secondary cases, might be included in a risk-factor analysis of the environment but excluded from an investigation of source predictors of transmission. Such approaches risk certain limitations. If, for example, the index and five secondary cases are included in the analysis of the environment, is one truly obtaining six independent measurements that can go into a statistical model? Or is the same environmental factor (eg, household or prison ward) being repeated six times? And if the secondary cases are excluded, how confident are we that the first case or the epidemiologically suspected index that we included is truly the one that initiated the outbreak? It is well established that an infant with tuberculosis triggers a source investigation to identify the adult with contagious tuberculosis; in a larger outbreak, the directionality of transmission may not always be apparent. For instance, in an apparent outbreak of 50 culture-confirmed tuberculosis cases in northern Canada, WGS identified six subgroups of clonal spread; in one, an individual brought to medical attention as a contact was later found to be a source case, based on cavitary, smear-positive disease and WGS data [64]. Given that WGS defines outbreak-related cases better than older strain typing modalities but also suggests a putative directionality of transmission (ie, based on the accumulation of SNPs relative to most common recent ancestor), it is possible that a refined understanding of the drivers of transmission in epidemiologically well-defined settings will emerge from the application of this technology. However, SNP-based definitions to support transmission in relatively short time scales are not well established and are likely to be contextually dependent (ie, high-incidence versus low-incidence areas).

A final consideration that has received limited attention is the characteristics of the inoculum. Classically, the most-contagious patients are those who cough and those with untreated, smear-positive and cavitary disease. As these parameters are readily estimated by history (cough and treatment) and relatively simple tests (microscopy and radiography), it remains practical to use them to help guide patient care, as well as a standard for estimating infectivity (however an insensitive tool it might be) to guide public health assessments, including in children, people with HIV infection, and patients with extrapulmonary disease (although the sensitivity of smear microscopy falls to <50% in these groups). But several questions exist. Are these independent indicators or merely correlated measures of the same process (ie, density of bacteria being spread from a patient)? Is the number of bacteria in the liquid phase of sputum an appropriate proxy for the number of bacteria expired in air from an individual with tuberculosis? Can we use genome equivalents from PCR-based assays to more accurately quantify bacterial burden in sputum, as a continuous rather than dichotomous variable? Answering these questions may additionally help to evaluate new therapies for treatment of tuberculosis.

## HETEROGENEITY OF TRANSMISSION: WHERE IS TUBERCULOSIS TRANSMITTED?

Several studies conducted in Africa have estimated the proportion of transmission occurring within households. In comparing genotypes of cases occurring within households or community settings, Verver et al estimated that <20% of infections were acquired within the household [65]. A study from South Africa used social contact diaries and portable CO_2_ monitors to infer exposures in a township with a high tuberculosis burden; an estimated 16% of infections occurred within households [32], which was confirmed by molecular epidemiologic findings [66]. Martinez et al performed a meta-analysis of studies reporting *M. tuberculosis* infection among children in households with and those without a household contact to estimate the population attributable fraction of household exposure [67]; their estimate of 14% (95% confidence interval, 11.6%–16.3%) was consistent with estimates from these other approaches in settings where the tuberculosis incidence is high [32, 66]. In addition, Glynn et al conducted surveillance for >13 years in a district in rural Malawi, using contact questionnaires and WGS; they found that a minority of tuberculosis cases could be attributed to infection from known contacts [68]. Overall, it appears that in medium-burden and high-burden areas, the majority of tuberculosis transmission occurs outside of households and may not necessarily be attributed to known close contacts; this suggests that the population-level impact of contact investigations, as a control measure in these areas, warrants further consideration (see the subsection “Areas of High Tuberculosis Incidence” below) [69]. Nevertheless, a regionally and culturally appropriate, balanced approach is needed; at the patient level, contact investigations are likely to yield a greater proportion of new tuberculosis cases than any other case finding approach.

However, determining where tuberculosis transmission specifically occurs outside of households is considerably more challenging. The potential for airborne transmission to occur even during brief exposures, combined with the variable periods of latency, make establishing specific tuberculosis transmission linkages exceptionally difficult. Despite these challenges, certain specific settings have been identified as substantial contributors to tuberculosis risk. Nosocomial transmission was a major driver of MDR-TB outbreaks in the United States, Spain, and Italy in the 1990s [70–73]; hospital-associated transmission was also responsible for the explosive dissemination of XDR-TB in a rural South African district in the mid-2000s [74]. Homeless shelters have been frequently identified as drivers of tuberculosis outbreaks [75]. Prisons have similarly been sources of not only incident outbreaks in low-burden countries [76] but also high levels of endemic transmission in low-income and middle-income countries [77]. Using national tuberculosis notification data from Brazil, Bourdillon et al found that >25% of all incident cases among men aged 20–29 years occurred within prisons, where notification rates were 31 times greater than in the general population [1, 78]. Other high-risk community settings implicated in tuberculosis transmission include public transportation [30], churches [31], schools [32, 33], bars [31], and slums [34–36].

The concentration of tuberculosis transmission in certain settings and subpopulations also leads to transmission heterogeneity, which can act to increase the effective reproductive number [79] and may make control of transmission more difficult. Basu et al described these settings as “institutional amplifiers” and demonstrated how they can increase incidence even when high levels of case detection and treatment success are established in the broader community [80]. By understanding the settings and groups at high risk for tuberculosis, however, we can more efficiently target interventions to achieve tuberculosis control. For example, Dowdy et al modeled data from Rio de Janeiro and demonstrated that targeting interventions to high-risk slum areas where 6% of the population resides could be as effective in reducing tuberculosis as equivalent measures conducted for the other 94% of the population [81].

### Areas of High Tuberculosis Incidence

In high-incidence areas, both epidemiologic and molecular studies have consistently shown that the majority of tuberculosis transmission does not come from known contacts [65, 66, 68, 82–87]. For example, in northern Malawi, estimates based on case-control studies, RFLP-based studies, and studies using WGS all concluded that the proportions of tuberculosis cases attributable to known contacts was <15% [68, 83, 84]. Perhaps most surprising was the finding that more than half of the individuals with tuberculosis who had contact with a smear-positive case in their household or in a close family member had a different strain than that contact, showing that this person was not the source of their infection [68, 84].

 These findings raise questions on the timing of infection(s) and where most infections are occurring. Work on the dynamics of aerosols suggests that infections are likely to be occurring mainly in indoor areas where air exchange is limited [4, 88]. This may include places where people are crowded together, although direct evidence is currently limited [66, 89]. Lack of ventilation may contribute to the high tuberculosis incidence in colder areas, such as the Western Cape of South Africa, and may have contributed to the spread of tuberculosis in Europe a century ago. It may also explain the paradoxical finding that tuberculosis incidence may be lower than expected among poorer families in rural Africa; they are less likely than those who are less poor to have glass windows, meet indoors, or use minibuses [90, 91]. In estimating setting-specific proportions of individuals with tuberculosis who transmit disease, the higher risk due to close, indoor contact needs to be balanced against the frequency of such contacts; more transmissions may occur from many low-risk contacts than from fewer high-risk ones [92].

Many areas of high tuberculosis incidence also have a high HIV prevalence. Clinics that provide HIV care can be potential amplifiers of tuberculosis epidemics when patients with undiagnosed tuberculosis mix with immunologically susceptible individuals. However, the effect of HIV itself on transmission appears to be more complex. Patients with tuberculosis who are infected with HIV are less likely to have smear-positive disease, and even those with smear-positive disease may transmit less than those who are HIV negative [66, 84]. This may be because they seek treatment or die earlier, shortening the duration of infectiousness. Nevertheless, because a high proportion of tuberculosis cases are HIV positive, they may contribute a large proportion of transmission events [84]. A study of tuberculosis among gold miners found that incidence increased following the HIV epidemic among HIV-negative and HIV-positive South African miners, showing that *M. tuberculosis* transmission increased overall [93]. Antiretroviral therapy, which reduces the risk of tuberculosis in HIV-infected individuals [94], has reduced tuberculosis incidence at the population level (at least in the short term) [95, 96]. However, even when antiretroviral therapy is available, transmission indices may remain high. A study in KwaZulu Natal, South Africa, a HIV endemic setting, found that despite high antiretroviral therapy coverage among XDR TB patients with HIV, upwards of 70% of cases were attributed to recent transmission [2]. Of note, a similar proportion (approximately 70%) of MDR-TB cases due to transmission was reported from Shanghai, China, where the prevalence of HIV infection is relatively low [1]. These studies of drug-resistant tuberculosis suggest that transmission is the driving force of tuberculosis incidence in areas of high and low HIV prevalence.

The effects of different genotypes on transmission can be studied in high-incidence areas when *M. tuberculosis* genotypes are not concentrated in particular population subgroups. For example, there is some evidence from contact and molecular clustering and network studies that lineage 2 (East-Asian/Beijing) and perhaps lineage 3 (East-African Indian) are more transmissible, whereas lineage 1 (Indo-Oceanic) and lineages 5 and 6 (the *Mycobacterium africanum* strains) are the least likely to transmit and cause disease [57, 97]. This fits with the wide geographical reach of Beijing strains [98], compared with the more limited distribution of *M. africanum*.

A major limitation to the study of transmission in high-incidence areas is the low sampling fraction (typically due to diagnostic delays or infrastructural limitations) and the representativeness of cases in the study to all infectious tuberculosis in the population. In these settings, the proportion of undiagnosed but diagnosable tuberculosis cases that contribute to transmission is likely substantial [7]. Finally, measurement of transmission is more challenging in high-burden settings, as incident outbreaks may be obscured by the background multiplicity of circulating strains.

### Areas of Low Tuberculosis Incidence

Key aspects of recent transmission, particularly measures of its relative size and scope, may be more readily discernible in low-incidence areas, where discrete chains of transmission can be identified or estimated. Furthermore, where resources are dedicated to universal genotyping of all culture-confirmed cases and tuberculosis molecular surveillance is formally established, additional insight is possible through the combined use of clinical, epidemiologic, and molecular data at the patient level [45, 46]. For example, the estimate that approximately 14% of United States cases are attributed to recent transmission is a valuable indicator. These proportions are markedly higher among US-born persons (27%), compared with non–US-born persons (8%). However, considerable demographic and geographic heterogeneity in these estimates also exists, implying that specific subpopulations might be prioritized for targeted intervention.

A key goal is to detect and interrupt transmission early enough to prevent incident genotypes from becoming epidemiologically entrenched in the population. In the United States, molecular surveillance [99] to detect and characterize clusters that may represent recent transmission has facilitated progress toward this goal [100]. In tandem, rapid uptake of technologies for WGS and phylogenetic analysis of select clusters that likely represent recent transmission has shown considerable promise in more precisely identifying outbreaks and outbreak-related cases. Thus, focused interventions to interrupt transmission and avoidance of unnecessary, resource-intensive investigations can often be effectively accomplished.

Even within low-incidence areas, however, high-incidence settings have been identified. Large clusters of prevalent genotypes can become entrenched due to prolonged, uncontrolled transmission within relatively closed populations of persons with limited access to clinical and public health services. Examples include long-standing outbreaks among urban homeless populations in major US cities [47] and impoverished rural settings with minority and indigenous populations [101]. In such settings, the ability to distinguish cases due to the reactivation of remotely acquired infection from cases of recent transmission with rapid progression to disease is often confounded by minimal strain diversity, even at the level of SNPs [102]. Thus, health officials must epidemiologically investigate local drivers of transmission so that prevention measures to find and treat latent *M. tuberculosis* infection and active case finding to characterize and interrupt transmission can be prioritized and accomplished effectively.

## DISCUSSION

**Figure 1.**
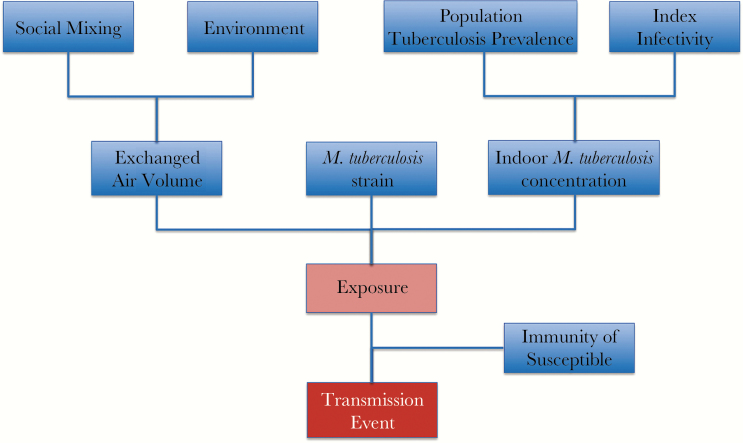
Factors influencing transmission.

We have presented evidence that the drivers of tuberculosis transmission in high-burden and low-burden settings appear markedly different. In high-burden settings, a large proportion of tuberculosis cases are due to recent transmission, and the burden of disease among infants and children is considerable. In contrast, the major proportion of tuberculosis in low-burden settings results from remote transmission, and the age distribution of tuberculosis is skewed toward older ages. In settings with lower burdens of tuberculosis, chains of transmission are more easily identified, whereas in settings with high tuberculosis burden, it is often more difficult to distinguish individual chains of transmission from the large quantity of background transmission. Institutional amplifiers, such as homeless shelters and prisons, may exist in both low-burden and high-burden settings. Within the extremes of tuberculosis burden, however, there remains considerable heterogeneity in transmission. It is possible therefore to view transmission as part of a continuum. In high-burden settings, endemic transmission results from a complex interplay between community and institutional amplifiers, resulting in generalized endemic tuberculosis. With improvement in general socioenvironmental factors, endemic tuberculosis continues to be transmitted in an increasingly smaller number of sites, resulting in more localized endemic tuberculosis restricted to subpopulations. As the proportion of the population affected by endemic transmission reduces further, it becomes easier to identify incident outbreaks, which often result from a highly infectious case in specific enabling environments. Thus, the expected transition in the nature of transmission would be a spectrum from long-standing (prevalent) outbreaks and endemic transmission occurring through institutional and community amplifiers, together with unrecognized, incident outbreaks occurring in higher-burden areas, to incident outbreaks increasingly being detected predominantly in lower-burden areas. A summary of key factors influencing transmission is depicted in Figure 1.

Owing to the relative ease of measuring tuberculosis transmission between contacts made within the confined household environment, household transmission and case notification rates in children have been used as end points to quantify the population-level impact of transmission control measures. However, household transmission in high-burden settings has been shown to represent a relatively small proportion of all tuberculosis transmissions. This may be because the highly associative mixing of a small number of household members does not necessarily reflect the actual transmission drivers resulting from an array of community and institutional tuberculosis amplifiers. Whereas household contact studies might reflect populations with relatively homogeneous tuberculosis transmission risk, the actual heterogeneity of tuberculosis transmission increases the complexity of the household-to-population transmission relationships. Transmission within institutions, such as homeless shelters and prisons, contribute significantly to population burden. Identification and control in sites with the highest transmission risks appears to provide a disproportionate benefit for tuberculosis control.

The tuberculosis caseload observed within subpopulations and settings may conflate estimates of recent transmission, including both infection and progression to disease risks. However, newer technologies can help distinguish true outbreaks from pseudo-outbreaks. For example, the use of proximity detection (eg, global positioning system mapping) or carbon dioxide monitoring has helped identify socioenvironmental hot spots of transmission, including public transportation, churches, schools, and bars [103]. Molecular methods, such as WGS, offer greatly improved resolution, compared with earlier genotyping methods, which have overestimated true transmission chains. Nonetheless, molecular methods remain limited to patients with culture-positive sputum specimens and still require interpretation within a framework of conventional epidemiology. Moreover, studies that rely on a passive case detection system are likely undersampling all of the infectious cases that remain undiagnosed but diagnosable [7, 104]. The contribution of such cases to tuberculosis incidence has yet to be determined.

**Figure 2.**
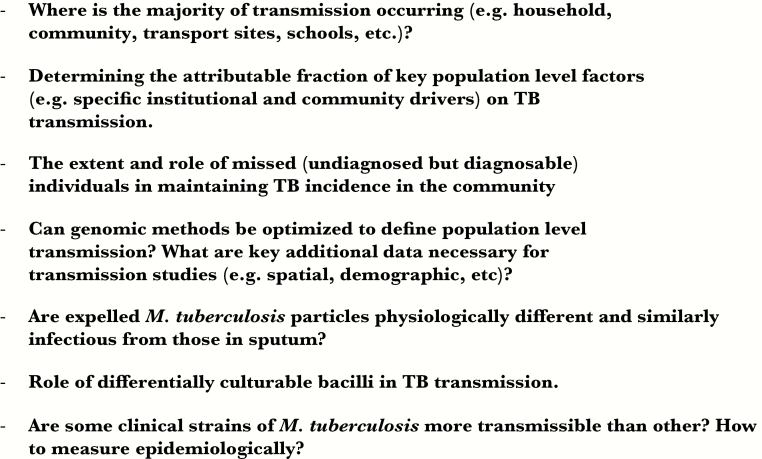
Some key questions relevant for transmission studies.

In summary, it is important to recognize the heterogeneity of tuberculosis transmission in determining effective control strategies for a population (Figure 1). While tuberculosis treatment has a survival benefit for the patient with tuberculosis, it can also decrease the prevalence of tuberculosis in a community. However, population-level tuberculosis control strategies to interrupt transmission need to be targeted at the overall transmission pathways that predominate. When endemic tuberculosis is widespread, the focus should be on identifying specific community and institutional tuberculosis amplifiers and hot spots of transmission. In localized endemic tuberculosis, case finding and environmental control measures should target highly burdened subpopulations. In incident outbreaks, early molecular identification, control of source cases with treatment for active disease, and identification of high risk contacts would be favored. Gaps in our knowledge of the factors driving transmission (Figure 2), including the contribution of the infective inoculum, the role of differentially culturable bacilli, the spectrum of infectiveness of tuberculosis sources, and how *M. tuberculosis* bacilli phenotypically adapt to survive outside the infected host, will need to be addressed.

## Notes

***Acknowledgments.***  We thank the workshop participants and session chairs, for valuable discussions; Dr N. Sarita Shah, for reviewing earlier versions of the manuscript; staff at the Division of AIDS, National Institute of Allergy and Infectious Disease, particularly Ms Kathleen Muldoon, for editorial and administrative assistance; and Zelda Moran, for technical assistance in manuscript preparation.

***Disclaimer.***  The views expressed in this article represents the authors opinion and not necessarily that of the National Allergy and Infectious Disease or National Institutes of Health.

***Financial support.*** This work is supported by the Division of AIDS, National Institute of Allergy and Infectious Disease, National Institute of Health, Department of Health and Human Services (contract HHSN272201100001G [Research Support Services for the Division of AIDS]); the Bill and Melinda Gates Foundation; the Centers for Disease Control and Prevention; and the South African Medical Research Council.

***Supplement sponsorship.*** This work is part of a supplement sponsored by the Centers for Disease Control and Prevention and the National Institutes of Health.

***Potential conflicts of interest.*** R. R. is contracted by Kelly Services, which receives federal funds through government contract with the National Institutes of Health. All other authors report no potential conflicts All authors have submitted the ICMJE Form for Disclosure of Potential Conflicts of Interest. Conflicts that the editors consider relevant to the content of the manuscript have been disclosed.

## References

[1] Yang C, Luo T, Shen X Transmission of multidrug-resistant Mycobacterium tuberculosis in Shanghai, China: a retrospective observational study using whole-genome sequencing and epidemiological investigation. Lancet Infect Dis 2017; 17:275–84. 27919643 10.1016/S1473-3099(16)30418-2 PMC5330813

[2] Shah NS, Auld SC, Brust JC Transmission of extensively drug-resistant tuberculosis in South Africa. N Engl J Med 2017; 376:243–53. 28099825 10.1056/NEJMoa1604544 PMC5330208

[3] Yates TA, Khan PY, Knight GM The transmission of Mycobacterium tuberculosis in high burden settings. Lancet Infect Dis 2016; 16:227–38. 26867464 10.1016/S1473-3099(15)00499-5

[4] Wood R, Morrow C, Barry CE 3rd Real-time investigation of tuberculosis transmission: developing the respiratory aerosol sampling chamber (RASC). PLoS One 2016; 11:e0146658. 26807816 10.1371/journal.pone.0146658 PMC4726558

[5] Fennelly KP, Martyny JW, Fulton KE, Orme IM, Cave DM, Heifets LB Cough-generated aerosols of Mycobacterium tuberculosis: a new method to study infectiousness. Am J Respir Crit Care Med 2004; 169:604–9. 14656754 10.1164/rccm.200308-1101OC

[6] Fennelly KP, Jones-López EC, Ayakaka I Variability of infectious aerosols produced during coughing by patients with pulmonary tuberculosis. Am J Respir Crit Care Med 2012; 186:450–7. 22798319 10.1164/rccm.201203-0444OC PMC3443801

[7] Dowdy DW, Basu S, Andrews JR Is passive diagnosis enough? The impact of subclinical disease on diagnostic strategies for tuberculosis. Am J Respir Crit Care Med 2013; 187:543–51. 23262515 10.1164/rccm.201207-1217OC PMC3733406

[8] Esmail H, Barry CE 3rd, Young DB, Wilkinson RJ The ongoing challenge of latent tuberculosis. Philos Trans R Soc Lond B Biol Sci 2014; 369:20130437. 24821923 10.1098/rstb.2013.0437 PMC4024230

[9] Esmail H, Lai RP, Lesosky M Characterization of progressive HIV-associated tuberculosis using 2-deoxy-2-[(18)F]fluoro-D-glucose positron emission and computed tomography. Nat Med 2016; 22:1090–3. 27595321 10.1038/nm.4161 PMC5055809

[10] Barry CE 3rd, Boshoff HI, Dartois V The spectrum of latent tuberculosis: rethinking the biology and intervention strategies. Nat Rev Microbiol 2009; 7:845–55. 19855401 10.1038/nrmicro2236 PMC4144869

[11] Storla DG, Yimer S, Bjune GA A systematic review of delay in the diagnosis and treatment of tuberculosis. BMC Public Health 2008; 8:15. 18194573 10.1186/1471-2458-8-15 PMC2265684

[12] Wells WF, Ratcliffe HL, Grumb C On the mechanics of droplet nuclei infection; quantitative experimental air-borne tuberculosis in rabbits. Am J Hyg 1948; 47:11–28. 18921435 10.1093/oxfordjournals.aje.a119179

[13] Riley RL, Wells WF, Mills CC, Nyka W, Mclean RL Air hygiene in tuberculosis: quantitative studies of infectivity and control in a pilot ward. Am Rev Tuberc 1957; 75:420–31. 13403171 10.1164/artpd.1957.75.3.420

[14] Kaplan G, Post FA, Moreira AL Mycobacterium tuberculosis growth at the cavity surface: a microenvironment with failed immunity. Infect Immun 2003; 71:7099–108. 14638800 10.1128/IAI.71.12.7099-7108.2003 PMC308931

[15] Jones-López EC, Acuña-Villaorduña C, Ssebidandi M Cough aerosols of Mycobacterium tuberculosis in the prediction of incident tuberculosis disease in household contacts. Clin Infect Dis 2016; 63:10–20. 27025837 10.1093/cid/ciw199 PMC5006106

[16] Jones-López EC, Namugga O, Mumbowa F Cough aerosols of Mycobacterium tuberculosis predict new infection: a household contact study. Am J Respir Crit Care Med 2013; 187:1007–15. 23306539 10.1164/rccm.201208-1422OC PMC3707366

[17] Harris TG, Sullivan Meissner J, Proops D Delay in diagnosis leading to nosocomial transmission of tuberculosis at a New York City health care facility. Am J Infect Control 2013; 41:155–60. 22750037 10.1016/j.ajic.2012.02.015

[18] Cheng S, Chen W, Yang Y Effect of diagnostic and treatment delay on the risk of tuberculosis transmission in Shenzhen, China: an observational cohort study, 1993–2010. PLoS One 2013; 8:e67516. 23826313 10.1371/journal.pone.0067516 PMC3694886

[19] Corbett EL, Steketee RW, ter Kuile FO, Latif AS, Kamali A, Hayes RJ HIV-1/AIDS and the control of other infectious diseases in Africa. Lancet 2002; 359:2177–87. 12090997 10.1016/S0140-6736(02)09095-5

[20] Jeon CY, Murray MB Diabetes mellitus increases the risk of active tuberculosis: a systematic review of 13 observational studies. PLoS Med 2008; 5:e152. 18630984 10.1371/journal.pmed.0050152 PMC2459204

[21] Lin HH, Ezzati M, Murray M Tobacco smoke, indoor air pollution and tuberculosis: a systematic review and meta-analysis. PLoS Med 2007; 4:e20. 17227135 10.1371/journal.pmed.0040020 PMC1769410

[22] Lönnroth K, Williams BG, Stadlin S, Jaramillo E, Dye C Alcohol use as a risk factor for tuberculosis - a systematic review. BMC Public Health 2008; 8:289. 18702821 10.1186/1471-2458-8-289 PMC2533327

[23] Menon S, Rossi R, Nshimyumukiza L, Wusiman A, Zdraveska N, Eldin MS Convergence of a diabetes mellitus, protein energy malnutrition, and TB epidemic: the neglected elderly population. BMC Infect Dis 2016; 16:361. 27456231 10.1186/s12879-016-1718-5 PMC4960905

[24] Fennelly KP, Jones-López EC Quantity and Quality of Inhaled Dose Predicts Immunopathology in Tuberculosis. Front Immunol 2015; 6:313. 26175730 10.3389/fimmu.2015.00313 PMC4484340

[25] Swaminathan S, Rekha B Pediatric tuberculosis: global overview and challenges. Clin Infect Dis 2010; 50(Suppl 3):S184–94. 20397947 10.1086/651490

[26] Cruz AT, Starke JR Pediatric tuberculosis. Pediatr Rev 2010; 31:13–25; quiz -6. 20048035 10.1542/pir.31-1-13

[27] World Health Organization (WHO). Global tuberculosis report 2015. Geneva, Switzerland: WHO, 2015.

[28] Hermans S, Boulle A, Caldwell J, Pienaar D, Wood R Temporal trends in TB notification rates during ART scale-up in Cape Town: an ecological analysis. J Int AIDS Soc 2015; 18:20240. 26411694 10.7448/IAS.18.1.20240 PMC4584214

[29] Broekmans JF, Migliori GB, Rieder HL; World Health Organization, International Union Against Tuberculosis and Lung Disease, and Royal Netherlands Tuberculosis Association Working Group. European framework for tuberculosis control and elimination in countries with a low incidence. Recommendations of the World Health Organization (WHO), International Union Against Tuberculosis and Lung Disease (IUATLD) and Royal Netherlands Tuberculosis Association (KNCV) Working Group. Eur Respir J 2002; 19:765–75. 11999007 10.1183/09031936.02.00261402

[30] Andrews JR, Morrow C, Wood R Modeling the role of public transportation in sustaining tuberculosis transmission in South Africa. Am J Epidemiol 2013; 177:556–61. 23423215 10.1093/aje/kws331 PMC3657527

[31] Murray EJ, Marais BJ, Mans G A multidisciplinary method to map potential tuberculosis transmission ‘hot spots’ in high-burden communities. Int J Tuberc Lung Dis 2009; 13:767–74. 19460255

[32] Andrews JR, Morrow C, Walensky RP, Wood R Integrating social contact and environmental data in evaluating tuberculosis transmission in a South African township. J Infect Dis 2014; 210:597–603. 24610874 10.1093/infdis/jiu138 PMC4133578

[33] Andrews JR, Hatherill M, Mahomed H The dynamics of QuantiFERON-TB gold in-tube conversion and reversion in a cohort of South African adolescents. Am J Respir Crit Care Med 2015; 191:584–91. 25562578 10.1164/rccm.201409-1704OC PMC4384770

[34] Wobudeya E, Lukoye D, Lubega IR, Mugabe F, Sekadde M, Musoke P Epidemiology of tuberculosis in children in Kampala district, Uganda, 2009–2010; a retrospective cross-sectional study. BMC public health 2015; 15:967. 26407719 10.1186/s12889-015-2312-2 PMC4582927

[35] Pereira AGL, Medronho RdA, Escosteguy CC, Valencia LIO, Magalhães MdAFM Spatial distribution and socioeconomic context of tuberculosis in Rio de Janeiro, Brazil. Revista de saude publica 2015; 49:1–8. 26270014 10.1590/S0034-8910.2015049005470 PMC4544397

[36] Koenig SP, Rouzier V, Vilbrun SC Tuberculosis in the aftermath of the 2010 earthquake in Haiti. Bull World Health Organ 2015; 93:498–502. 26170508 10.2471/BLT.14.145649 PMC4490810

[37] Stýblo K, Meijer J, Sutherland I The transmission of tubercle bacilli: its trend in a human population. Bull World Health Organ 1969; 41:137–78. 5309081 PMC2427401

[38] Odhiambo J, Borgdorff M, Kiambih F Tuberculosis and the HIV epidemic: increasing annual risk of tuberculous infection in Kenya, 1986–1996. Am J Public Health 1999; 89:1078–82. 10394319 10.2105/ajph.89.7.1078 PMC1508825

[39] Kritzinger FE, den Boon S, Verver S No decrease in annual risk of tuberculosis infection in endemic area in Cape Town, South Africa. Trop Med Int Health 2009; 14:136–42. 19236665 10.1111/j.1365-3156.2008.02213.x

[40] Sutherland I, Bleiker M, Meijer J, Stýblo K The risk of tuberculous infection in the Netherlands from 1967 to 1979. Tubercle 1983; 64:241–53. 6606884 10.1016/0041-3879(83)90021-1

[41] Nagelkerke N, Heisterkamp S, Borgdorff M, Broekmans J, Van Houwelingen H Semi-parametric estimation of age-time specific infection incidence from serial prevalence data. Stat Med 1999; 18:307–20. 10070676 10.1002/(sici)1097-0258(19990215)18:3<307::aid-sim15>3.0.co;2-z

[42] van Embden JD, Cave MD, Crawford JT Strain identification of Mycobacterium tuberculosis by DNA fingerprinting: recommendations for a standardized methodology. J Clin Microbiol 1993; 31:406–9. 8381814 10.1128/jcm.31.2.406-409.1993 PMC262774

[43] Kasaie P, Mathema B, Kelton WD, Azman AS, Pennington J, Dowdy DW A novel tool improves existing estimates of recent tuberculosis transmission in settings of sparse data collection. PLoS One 2015; 10:e0144137. 26679499 10.1371/journal.pone.0144137 PMC4683006

[44] Small PM, Hopewell PC, Singh SP The epidemiology of tuberculosis in San Francisco. A population-based study using conventional and molecular methods. N Engl J Med 1994; 330:1703–9. 7910661 10.1056/NEJM199406163302402

[45] France AM, Grant J, Kammerer JS, Navin TR A field-validated approach using surveillance and genotyping data to estimate tuberculosis attributable to recent transmission in the United States. Am J Epidemiol 2015; 182:799–807. 26464470 10.1093/aje/kwv121 PMC5996379

[46] Yuen CM, Kurbatova EV, Click ES, Cavanaugh JS, Cegielski JP Association between Mycobacterium tuberculosis complex phylogenetic lineage and acquired drug resistance. PLoS One 2013; 8:e83006. 24376623 10.1371/journal.pone.0083006 PMC3871645

[47] Bamrah S, Yelk Woodruff RS, Powell K, Ghosh S, Kammerer JS, Haddad MB Tuberculosis among the homeless, United States, 1994–2010. Int J Tuberc Lung Dis 2013; 17:1414–9. 24125444 10.5588/ijtld.13.0270 PMC5077150

[48] Valença MS, Scaini JL, Abileira FS, Gonçalves CV, von Groll A, Silva PE Prevalence of tuberculosis in prisons: risk factors and molecular epidemiology. Int J Tuberc Lung Dis 2015; 19:1182–7. 26459530 10.5588/ijtld.15.0126

[49] Sacchi FP, Praça RM, Tatara MB Prisons as reservoir for community transmission of tuberculosis, Brazil. Emerg Infect Dis 2015; 21:452–5. 25642998 10.3201/eid2103.140896 PMC4344267

[50] Walker TM, Lalor MK, Broda A Assessment of Mycobacterium tuberculosis transmission in Oxfordshire, UK, 2007-12, with whole pathogen genome sequences: an observational study. Lancet Respir Med 2014; 2:285–92. 24717625 10.1016/S2213-2600(14)70027-X PMC4571080

[51] Rieder HL. Epidemiologic basis of tuberculosis control. Paris, France: International Union Against Tuberculosis and Lung Disease, 1999.

[52] Bifani PJ, Plikaytis BB, Kapur V Origin and interstate spread of a New York City multidrug-resistant Mycobacterium tuberculosis clone family. JAMA 1996; 275:452–7. 8627966

[53] Mathema B, Kurepina NE, Bifani PJ, Kreiswirth BN Molecular epidemiology of tuberculosis: current insights. Clin Microbiol Rev 2006; 19:658–85. 17041139 10.1128/CMR.00061-05 PMC1592690

[54] Roetzer A, Diel R, Kohl TA Whole genome sequencing versus traditional genotyping for investigation of a *Mycobacterium tuberculosis* outbreak: a longitudinal molecular epidemiological study. PLoS Med 2013; 10:e1001387. 23424287 10.1371/journal.pmed.1001387 PMC3570532

[55] Walker TM, Ip CL, Harrell RH Whole-genome sequencing to delineate Mycobacterium tuberculosis outbreaks: a retrospective observational study. Lancet Infect Dis 2013; 13:137–46. 23158499 10.1016/S1473-3099(12)70277-3 PMC3556524

[56] Lieberman TD, Wilson D, Misra R Genomic diversity in autopsy samples reveals within-host dissemination of HIV-associated Mycobacterium tuberculosis. Nat Med 2016; 22:1470–4. 27798613 10.1038/nm.4205 PMC5508070

[57] Guerra-Assuncao JA, Crampin AC, Houben RM Large-scale whole genome sequencing of M. tuberculosis provides insights into transmission in a high prevalence area. Elife 2015; 4. 10.7554/eLife.05166 PMC4384740 25732036

[58] Ford CB, Shah RR, Maeda MK Mycobacterium tuberculosis mutation rate estimates from different lineages predict substantial differences in the emergence of drug-resistant tuberculosis. Nat Genet 2013; 45:784–90. 23749189 10.1038/ng.2656 PMC3777616

[59] Bryant JM, Schürch AC, van Deutekom H Inferring patient to patient transmission of Mycobacterium tuberculosis from whole genome sequencing data. BMC Infect Dis 2013; 13:110. 23446317 10.1186/1471-2334-13-110 PMC3599118

[60] O’Neill MB, Mortimer TD, Pepperell CS Diversity of Mycobacterium tuberculosis across evolutionary scales. PLoS Pathog 2015; 11:e1005257. 26562841 10.1371/journal.ppat.1005257 PMC4642946

[61] Didelot X, Gardy J, Colijn C Bayesian inference of infectious disease transmission from whole-genome sequence data. Mol Biol Evol 2014; 31:1869–79. 24714079 10.1093/molbev/msu121 PMC4069612

[62] Didelot X, Fraser C, Gardy J, Colijn C Genomic infectious disease epidemiology in partially sampled and ongoing outbreaks. Mol Biol Evol 2017; 34:997–1007. 28100788 10.1093/molbev/msw275 PMC5850352

[63] Chengalroyen MD, Beukes GM, Gordhan BG Detection and quantification of differentially culturable tubercle bacteria in sputum from patients with tuberculosis. Am J Respir Crit Care Med 2016; 194:1532–40. 27387272 10.1164/rccm.201604-0769OC PMC5215032

[64] Lee RS, Radomski N, Proulx JF Reemergence and amplification of tuberculosis in the Canadian arctic. J Infect Dis 2015; 211:1905–14. 25576599 10.1093/infdis/jiv011

[65] Verver S, Warren RM, Munch Z Proportion of tuberculosis transmission that takes place in households in a high-incidence area. Lancet 2004; 363:212–4. 14738796 10.1016/S0140-6736(03)15332-9

[66] Middelkoop K, Mathema B, Myer L Transmission of tuberculosis in a South African community with a high prevalence of HIV infection. J Infect Dis 2015; 211:53–61. 25053739 10.1093/infdis/jiu403 PMC4334823

[67] Martinez L, Shen Y, Mupere E, Kizza A, Hill PC, Whalen CC Transmission of Mycobacterium tuberculosis in households and the community: a systematic review and meta-analysis. Am J Epidemiol 2017; 185:1327–39. 28982226 10.1093/aje/kwx025 PMC6248487

[68] Glynn JR, Guerra-Assunção JA, Houben RM Whole genome sequencing shows a low proportion of tuberculosis disease is attributable to known close contacts in rural Malawi. PLoS One 2015; 10:e0132840. 26181760 10.1371/journal.pone.0132840 PMC4504505

[69] Kasaie P, Andrews JR, Kelton WD, Dowdy DW Timing of tuberculosis transmission and the impact of household contact tracing. An agent-based simulation model. Am J Respir Crit Care Med 2014; 189:845–52. 24559425 10.1164/rccm.201310-1846OC

[70] Control CfD Nosocomial transmission of multidrug-resistant tuberculosis among HIV-infected persons--Florida and New York, 1988–1991. MMWR Morb Mortal Wkly Rep 1991; 40:585. 1870559

[71] Frieden TR, Sherman LF, Maw KL A multi-institutional outbreak of highly drug-resistant tuberculosis: epidemiology and clinical outcomes. JAMA 1996; 276:1229–35. 8849750

[72] Rullán JV, Herrera D, Cano R Nosocomial transmission of multidrug-resistant Mycobacterium tuberculosis in Spain. Emerg Infect Dis 1996; 2: 125–9. 8903213 10.3201/eid0202.960208 PMC2639835

[73] Moro ML, Gori A, Errante I An outbreak of multidrug-resistant tuberculosis involving HIV-infected patients of two hospitals in Milan, Italy. Italian Multidrug-Resistant Tuberculosis Outbreak Study Group. AIDS 1998; 12:1095–102. 9662207

[74] Gandhi NR, Weissman D, Moodley P Nosocomial transmission of extensively drug-resistant tuberculosis in a rural hospital in South Africa. J Infect Dis 2013; 207:9–17. 23166374 10.1093/infdis/jis631 PMC3523793

[75] Nardell E, McInnis B, Thomas B, Weidhaas S Exogenous reinfection with tuberculosis in a shelter for the homeless. N Engl J Med 1986; 315:1570–5. 3097543 10.1056/NEJM198612183152502

[76] Valway SE, Greifinger RB, Papania M Multidrug-resistant tuberculosis in the New York State prison system, 1990–1991. J Infect Dis 1994; 170:151–6. 8014491 10.1093/infdis/170.1.151

[77] Baussano I, Williams BG, Nunn P, Beggiato M, Fedeli U, Scano F Tuberculosis incidence in prisons: a systematic review. PLoS Med 2010; 7:e1000381. 21203587 10.1371/journal.pmed.1000381 PMC3006353

[78] Bourdillon PM, Gonçalves CC, Pelissari DM Increase in tuberculosis cases among prisoners, Brazil, 2009-2014(1). Emerg Infect Dis 2017; 23:496–9. 28221118 10.3201/eid2303.161006 PMC5382752

[79] Andrews JR, Basu S, Dowdy DW, Murray MB [The epidemiological advantage of preferential targeting of tuberculosis control at the poor]. Rev Panam Salud Publica 2015; 38:186–94. 26757996

[80] Basu S, Stuckler D, McKee M Addressing institutional amplifiers in the dynamics and control of tuberculosis epidemics. Am J Trop Med Hyg 2011; 84:30–7. 21212197 10.4269/ajtmh.2011.10-0472 PMC3005502

[81] Dowdy DW, Golub JE, Chaisson RE, Saraceni V Heterogeneity in tuberculosis transmission and the role of geographic hotspots in propagating epidemics. Proc Natl Acad Sci U S A 2012; 109:9557–62. 22645356 10.1073/pnas.1203517109 PMC3386125

[82] Nair SS, Ramanath Rao G, Chandrasekhar P Distribution of tuberculosis infection and disease in clusters in rural households. Ind J Tuberc 1971; 18:3–9.

[83] Crampin AC, Glynn JR, Floyd S Tuberculosis and gender: exploring the patterns in a case control study in Malawi. Int J Tuberc Lung Dis 2004; 8:194–203. 15139448

[84] Crampin AC, Glynn JR, Traore H Tuberculosis transmission attributable to close contacts and HIV status, Malawi. Emerg Infect Dis 2006; 12: 729–35. 16704828 10.3201/eid1205.050789 PMC3374426

[85] Wilkinson D, Pillay M, Crump J, Lombard C, Davies GR, Sturm AW Molecular epidemiology and transmission dynamics of Mycobacterium tuberculosis in rural Africa. Trop Med Int Health 1997; 2:747–53. 9294544 10.1046/j.1365-3156.1997.d01-386.x

[86] Buu TN, van Soolingen D, Huyen MN Tuberculosis acquired outside of households, rural Vietnam. Emerg Infect Dis 2010; 16:1466–8. 20735935 10.3201/eid1609.100281 PMC3294980

[87] Brooks-Pollock E, Becerra MC, Goldstein E, Cohen T, Murray MB Epidemiologic inference from the distribution of tuberculosis cases in households in Lima, Peru. J Infect Dis 2011; 203:1582–9. 21592987 10.1093/infdis/jir162 PMC3096792

[88] Wood R, Morrow C, Ginsberg S Quantification of shared air: a social and environmental determinant of airborne disease transmission. PLoS One 2014; 9:e106622. 25181526 10.1371/journal.pone.0106622 PMC4152288

[89] Chamie G, Wandera B, Marquez C Identifying locations of recent TB transmission in rural Uganda: a multidisciplinary approach. Trop Med Int Health 2015; 20:537–45. 25583212 10.1111/tmi.12459 PMC4355181

[90] Odone A, Crampin AC, Mwinuka V Association between socioeconomic position and tuberculosis in a large population-based study in rural Malawi. PLoS One 2013; 8:e77740. 24204945 10.1371/journal.pone.0077740 PMC3804525

[91] Chamie G, Wandera B, Luetkemeyer A Household ventilation and tuberculosis transmission in Kampala, Uganda. Int J Tuberc Lung Dis 2013; 17:764–70. 23676159 10.5588/ijtld.12.0681 PMC13218450

[92] Rose G Sick individuals and sick populations. Int J Epidemiol 1985; 14:32–8. 3872850 10.1093/ije/14.1.32

[93] Glynn JR, Murray J, Bester A, Nelson G, Shearer S, Sonnenberg P Effects of duration of HIV infection and secondary tuberculosis transmission on tuberculosis incidence in the South African gold mines. AIDS 2008; 22:1859–67. 18753936 10.1097/QAD.0b013e3283097cfa

[94] Lawn SD, Badri M, Wood R Tuberculosis among HIV-infected patients receiving HAART: long term incidence and risk factors in a South African cohort. AIDS 2005; 19:2109–16. 16284460 10.1097/01.aids.0000194808.20035.c1

[95] Zachariah R, Bemelmans M, Akesson A Reduced tuberculosis case notification associated with scaling up antiretroviral treatment in rural Malawi. Int J Tuberc Lung Dis 2011; 15:933–7. 21682967 10.5588/ijtld.10.0666

[96] Middelkoop K, Wood R, Bekker LG The impact of antiretroviral treatment programs on tuberculosis notification rates. Int J Tuberc Lung Dis 2011; 15:1714; author reply -5. 10.5588/ijtld.11.0545 22118185

[97] de Jong BC, Hill PC, Aiken A Progression to active tuberculosis, but not transmission, varies by Mycobacterium tuberculosis lineage in The Gambia. J Infect Dis 2008; 198:1037–43. 18702608 10.1086/591504 PMC2597014

[98] Glynn JR, Kremer K, Borgdorff MW, Pujades Rodriguez M, van Soolingen D; European Concerted Action on New Generation Genetic Markers and Techniques for the Epidemiology and Control of Tuberculosis Beijing/W genotype Mycobacterium tuberculosis and drug resistance. Emerg Infect Dis 2006; 12:736–43.16704829

[99] Ghosh S, Moonan PK, Cowan L, Grant J, Kammerer S, Navin TR Tuberculosis genotyping information management system: enhancing tuberculosis surveillance in the United States. Infect Genet Evol 2012; 12:782–8. 22044522 10.1016/j.meegid.2011.10.013

[100] Teeter LD, Vempaty P, Nguyen DT; Tuberculosis Epidemiologic Studies Consortium. Validation of genotype cluster investigations for Mycobacterium tuberculosis: application results for 44 clusters from four heterogeneous United States jurisdictions. BMC Infect Dis 2016; 16:594. 27769182 10.1186/s12879-016-1937-9 PMC5075185

[101] Tollefson D, Bloss E, Fanning A, Redd JT, Barker K, McCray E Burden of tuberculosis in indigenous peoples globally: a systematic review. Int J Tuberc Lung Dis 2013; 17:1139–50. 23823137 10.5588/ijtld.12.0385 PMC6057791

[102] Lee RS, Radomski N, Proulx JF Population genomics of Mycobacterium tuberculosis in the Inuit. Proc Natl Acad Sci U S A 2015; 112:13609–14. 26483462 10.1073/pnas.1507071112 PMC4640744

[103] Andrews JR, Morrow C, Walensky RP, Wood R Integrating social contact and environmental data in evaluating tuberculosis transmission in a South African township. J Infect Dis 2014; 210:597–603. 24610874 10.1093/infdis/jiu138 PMC4133578

[104] Wood R, Middelkoop K, Myer L Undiagnosed tuberculosis in a community with high HIV prevalence: implications for tuberculosis control. Am J Respir Crit Care Med 2007; 175:87–93. 16973982 10.1164/rccm.200606-759OC PMC1899262

